# Decreased hernia recurrence using autologous platelet-rich plasma (PRP) with Strattice™ mesh in a rodent ventral hernia model

**DOI:** 10.1007/s00464-015-4645-4

**Published:** 2015-11-17

**Authors:** Jeffrey Van Eps, Joseph Fernandez-Moure, Fernando Cabrera, Xin Wang, Azim Karim, Bruna Corradetti, Paige Chan, Brian Dunkin, Ennio Tasciotti, Bradley Weiner, Warren Ellsworth

**Affiliations:** Department of Surgery, Houston Methodist Hospital, Houston, TX 77030 USA; Surgical Advanced Technologies Lab, Center for Regenerative Medicine, Houston Methodist Research Institute, Houston, TX 77030 USA; Department of Life and Environmental Sciences, Università Politecnica delle Marche, Via Brecce Bianche, 60131 Ancona, Italy; Methodist Institute for Technology, Innovation, and Education (MITIE), Houston Methodist Research Institute, Houston, TX 77030 USA; Weill Cornell Medical College, Cornell University, New York, NY 10021 USA; Houston Methodist Orthopedics and Sports Medicine, Houston Methodist Hospital, Houston, TX 77030 USA

**Keywords:** Platelet-rich plasma, Hernia, Strattice, Ventral hernia repair, Recurrence, Regeneration

## Abstract

**Background:**

Recurrence after ventral hernia repair (VHR) remains a multifactorial problem still plaguing surgeons today. Some of the many contributing factors include mechanical strain, poor tissue-mesh integration, and degradation of matrices. The high recurrence rate witnessed with the use of acellular dermal matrices (ADM) for definitive hernia repair has reduced their use largely to bridging repair and breast reconstruction. Modalities that improve classic cellular metrics of successful VHR could theoretically result in improved rates of hernia recurrence; autologous platelet-rich plasma (PRP) may represent one such tool, but has been underinvestigated for this purpose.

**Methods:**

Lewis rats (32) had chronic ventral hernias created surgically and then repaired with Strattice™ mesh alone (control) or mesh + autologous PRP. Samples were harvested at 3 and 6 months postoperatively and compared for gross, histologic, and molecular outcomes of: neovascularization, tissue incorporation, peritoneal adhesions, hernia recurrence, and residual mesh thickness.

**Results:**

Compared to control at 3 months postoperatively, PRP-treated rats displayed significantly more neovascularization of implanted mesh and considerable upregulation of both angiogenic genes (vEGF 2.73-fold, vWF 2.21-fold) and myofibroblastic genes (αSMA 9.68-fold, FSP-1 3.61-fold, Col1a1 3.32-fold, Col31a1 3.29-fold). Histologically, they also showed enhanced tissue deposition/ingrowth and diminished chronic immune cell infiltration. Peritoneal adhesions were less severe at both 3 (1.88 vs. 2.94) and 6 months (1.63 vs. 2.75) by Modified Hopkins Adhesion Scoring. PRP-treated rats experienced decreased hernia recurrence at 6 months (0/10 vs. 7/10) and had significantly improved ADM preservation as evidenced by quantification of residual mesh thickness.

**Conclusions:**

PRP is an autologous source of pro-regenerative growth factors and chemokines uniquely suited to soft tissue wound healing. When applied to a model of chronic VHR, it incites enhanced angiogenesis, myofibroblast recruitment and tissue ingrowth, ADM preservation, less severe peritoneal adhesions, and diminished hernia recurrence. We advocate further investigation regarding PRP augmentation of human VHR.

Ventral hernia repair (VHR) remains one of the most common surgical procedures performed in the USA today, with over 350,000 cases annually [1]. Although surgical indications vary, the majority of these are incisional (chronic) hernia repairs, as the risk of developing a chronic hernia after prior abdominal visceral surgery ranges from 2 to 20 % depending on surgical approach [2–4]. However, sanctioned standards of care are lacking in VHR and both prosthetic materials and repair techniques vary greatly according to individual providers and institutions. Additionally, despite the large clinical impact of these operations and their complications, the paucity of VHR research support available limits both a comprehensive understanding of the mechanisms involved and innovation upon current treatment modalities [5]. The use of prosthetic mesh is more than doubled from the mid-1980s until the new millennium, and although the advent of prosthetic VHR has allowed a 24–50 % reduction in recurrence rates compared to primary tissue repair, a multitude of new complications related to prosthetic material and local host microenvironment similarly arose [6–9]. For synthetic prostheses, these may include mesh extrusion, contraction, infection, erosion, and fistula formation; meanwhile, biologic prostheses such as human or porcine acellular dermal matrices (ADM) are criticized for their high mesh failure rates due to insufficient tissue incorporation or immunologic/enzymatic destruction [10–17].

Improvements in the local tissue response after implantation, either by improved mesh incorporation or by prevention of mesh bacterial colonization and/or degradation, could theoretically diminish such complications and enhance prosthetic VHR. Early cellularization of biosynthetic implants is posited to be protective from bacterial colonization and subsequent clinically significant infection [18]. Likewise, early angiogenesis and cellular population of ADMs prevent encapsulation or contamination events that are known to predispose them to mechanical failure and hernia recurrence [19, 20]. Supplementing VHR with synthetic growth factor/chemokine products has been investigated for this purpose and demonstrated ability to significantly augment the host tissue response, increase mechanical strength, and diminish incidence of incisional hernia formation [21–23]. Platelet-rich plasma (PRP) is a universally available, autologous product containing an array of growth factors and chemokines that are uniquely suited for amplifying soft tissue wound repair by boosting the proliferative wound healing phase, including: vEGF, PDGF, FGF, TGF-β, and SDF-1α. In prior studies, these components have been well characterized and proven sufficient for improved stromal cell migration/proliferation, angiogenesis, and extracellular matrix deposition, that translates to improved wound healing and enhanced tissue regeneration in models utilizing it alone or in combination with synthetic matrices [24–30]. Despite its attractive composition for musculoskeletal healing, PRP has been underutilized for purposes of VHR research or clinical enhancement. The aim of this study was to prove or disprove our existent hypothesis that the addition of autologous PRP to VHR with a non-cross-linked, porcine ADM would incite cellular events such as enhanced neovascularization and myofibroblast recruitment/infiltration that would ultimately result in decreased incidence of long-term mesh failure and hernia recurrence via preserved mesh integrity.

## Materials and methods

### Study design

To investigate the effect of autologous PRP in vivo, 32 adult male Lewis rats (Charles River Labs, Houston, TX) were randomly assigned to either a control (mesh alone) or experimental (mesh + PRP) group. Eight additional rats were utilized as blood donors for PRP isolation. Animal subjects were cared for postoperatively until killing at one of the two predetermined time points—3 or 6 months. Both groups had 6 animals/group assigned to the 3-month time point and 10 animals/group assigned to the 6-month time point—more animals were pre-emptively utilized at the outset for this long-term time point in case some animals suffered complication requiring exclusion from the study, which did not occur. Animal tissue was harvested at the above time points for histologic, molecular (10 days and 3 month samples), and gross evaluation. Primary endpoints of comparison between groups included: degree of neovascularization and collagen production at 3 months, expression of myofibroblastic genes at 3 months, peritoneal adhesion severity, residual mesh thickness at 6 months, and incidence of hernia recurrence at 6 months. All animal work was performed at Houston Methodist Research Institute (HMRI) under approval and supervision of the Institutional Animal Care and Use Committee (IACUC), and all investigators complied with the National Research Council’s *Guide for the Care and Use of Laboratory Animals*. Rats received water and chow ad libitum and were housed in pairs at Houston Methodist Research Institute (HMRI) until the study period began and only after the required 48 h of acclimation time had passed. Postsurgically rats were housed individually and monitored daily for weight loss or other complication for the first five postoperative days, then biweekly for 3 weeks, and finally weekly until the animal is killed. All animals were humanely euthanized by inhaled carbon dioxide gas followed by confirmatory bilateral thoracotomy prior to tissue harvest.

### PRP isolation, quantification, and activation

One particular reason for using Lewis strain rodents in this study is their inbred genetic background. This is advantageous for isolation of autologous PRP, as its components are not recognized by other recipients as foreign. Eight animals were utilized solely for PRP harvesting by collecting the available intravascular blood volume via terminal intracardiac blood draw under deep anesthesia. Briefly, anesthesia was induced via inhaled isoflurane/oxygen mixture at a concentration of 4.5–5 % within a plastic enclosure, followed by maintenance of deep anesthesia at 2.5–4 % delivered via nosecone. While supine with the anterior chest fur clipped, the chest wall was sterilized and draped in standard sterile fashion, and the chest cavity was entered sharply at the level of the xyphoid followed by median sternotomy. The ventricular cavity of the heart was punctured with a 18- or 21-gauge needle connected to a 12-mL syringe pre-filled with 1–2 mL of acid citrate dextrose (ACD) anticoagulant and the intravascular blood volume aspirated slowly. Typical yield of blood volume from a single rat was 8–12 mL. Though the speeds of each spin are variable in the literature, PRP was isolated from this whole-blood sample via a double-centrifugation technique as previously reported. First, whole blood was spun at 200 g for 15 min to isolate the plasma fractions. After removal of the red blood cell (RBC) and buffy coat components, the remaining plasma was centrifuged again at 1600 g for 10 min to pellet the platelets and separate from platelet-poor plasma. The total number of platelets was quantified using a Multisizer Coulter Counter (Beckman Coulter, Pasadena, CA) and the appropriate dilution prepared to generate a strictly standardized final dose concentration of 10^6^ platelets per microliter of plasma in the therapeutic PRP delivered [31]. Prior to surgical application for each experimental animal, an effective dose of 200 µL of PRP (2 × 10^8^ plt) was activated using bovine thrombin (1000U/mL, Sigma-Aldrich, St. Louis, MO) as previously described, although subsequent interaction with both exposed collagen I and tissue factor in the surgical wound, along with platelet coagulation itself, is also known to also stimulate platelet coagulation and activation [26, 27, 32–34].

### Surgical technique

**Fig. 1:**
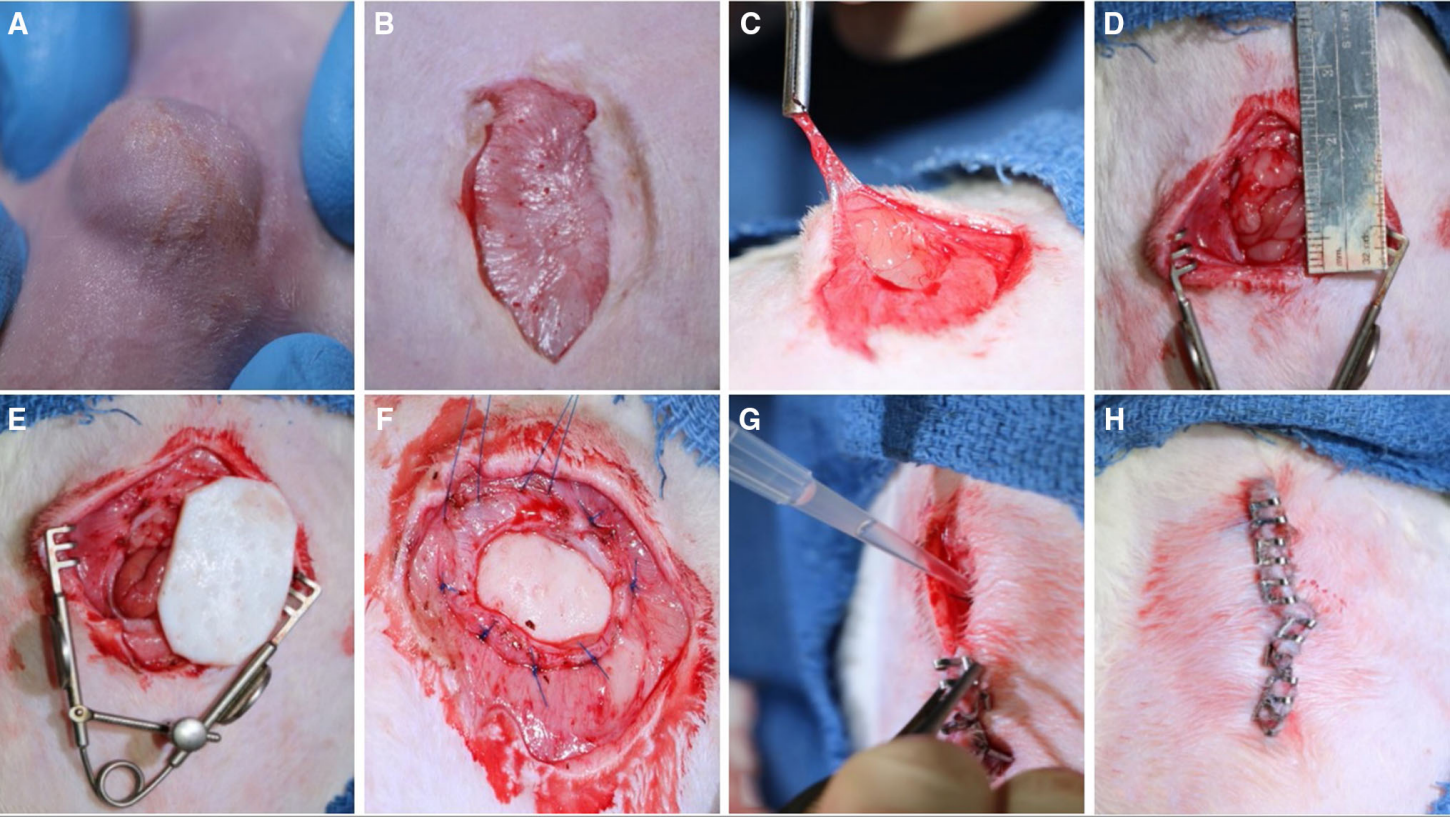
Surgical technique. Rodents had chronic ventral abdominal hernias created (**A**) by full-thickness incision of the linea alba, closure of the overlying skin, and waiting for ≥30 days. Chronic VHR was performed in bridging fashion using Strattice™ mesh via the same incision (**B**–**F**). Experimental rats had autologous PRP applied to the mesh surface at the time of implantation (**G**) prior to skin closure (**H**)

For preoperative anesthesia, all rats received buprenorphine (0.05 mg/kg) and carprofen (5 mg/kg) injected subcutaneously. After induction, anesthesia was maintained using a 2.5–3.0 % isoflurane/oxygen mixture via nosecone with the animal supine. Before sterile draping, the abdominal fur was clipped and the skin sterilized with three alternating scrubs using chlorhexidine gluconate and 70 % alcohol, and aseptic technique was maintained throughout the duration of the surgery. All animals first had a chronic ventral hernia created that was allowed to mature for a minimum of 30 days prior to secondary ventral hernia repair. This was accomplished by making a 3-cm (cm) vertical skin incision down to the abdominal wall musculature, followed by a 2-cm full-thickness incision of the abdominal wall at the linea alba followed by closure of the overlying skin using wound clips. All animals had Elizabethan collars (Kent Scientific, Torrington, CT) in place to prevent incisional chewing and evisceration for the first four postoperative days after both hernia creation and repair operations. Wound clips were removed on postoperative day 10. After the requisite time for hernia maturation had passed, VHR was performed using identical anesthesia modalities as above and via the prior skin incision. Using sharp dissection and electrocautery sparingly, the hernia sac was excised and the hernia borders defined (Fig. 1). After soaking in sterile saline for the recommended time per manufacturer, a piece of Strattice™ mesh cut into an ellipse was sutured into place in bridging intraperitoneal fashion with at least a 1.5-cm overlap of mesh from the hernia edge using eight interrupted 3–0 Prolene^®^ (Ethicon, Somerville, NJ) sutures (Fig. 1). Experimental animals had their mesh soaked briefly (10 min) in activated PRP prior to implantation, followed by application of the remainder of PRP to the mesh surface after suture fixation and prior to incisional closure with wound clips.

### Adhesion severity scoring

Similar to previous studies, we utilized a Modified Hopkins Adhesion Score to assess the number and severity of peritoneal adhesions encountered at the time of necropsy and specimen harvest [35, 36]. A numerical score from 0 to 4 was assigned with equal weighting to five different parameters of adhesion formation—frequency (number of bands), size/width, density (ranging from thin and transparent to opaque and dense), and difficulty of dissection (based on technique required and inadvertent serosal/organ injury, Table 1). Mean scores and standard deviation were calculated for each group at 3 and 6 months.

**Table 1:** Modified Hopkins Adhesion Score, adapted from Dubcenco et al. [35]

Score	Frequency	Size/width	Density	Dissection
0	0	No adhesions (cm)	No adhesions	No adhesions
1	1	<1	Single thin, filmy adhesion	Minimal blunt dissection, tears easily
2	2–3	1–2	Multiple thin, filmy adhesions	Blunt dissection only
3	3–4	2–3	Dense adhesion(s) with or without filmy adhesions	Sharp dissection or electrocautery, no organ/serosal damage
4	4+	3+	Matted adhesion(s) with or without filmy adhesions	Sharp dissection or electrocautery, with unavoidable organ/serosal damage

### Assessment of residual mesh thickness and hernia recurrence

Like previously performed historically, the native Strattice™ mesh utilized for this study was measured manually using a digital caliper at multiple locations after the recommended 2-min rehydration soak to obtain a baseline thickness value pre-implantation, which would later be compared to values obtained at our 6-month time point [37, 38]. With the implant cut in half at its midpoint to facilitate caliper measurement and histopathological processing, samples were harvesting with sharp dissection 6 months postoperatively. If mesh failure was present, the recorded value at that site (anatomic right, left, or middle mesh) was recorded as zero. Incidence of hernia recurrence was observed grossly at the time of necropsy and recorded for both groups.

### Histopathology

To visualize tissue incorporation and neovascularization differences, mesh samples with their tissue interface were harvested for histopathological processing and evaluation. At the time of necropsy, the entire mesh implant was circumferentially excised from the adherent abdominal wall by sharp dissection and transected at its midpoint. One half of the specimen was preserved in RNAlater (Life Technologies, Carlsbad, CA) according to manufacturers instruction for subsequent molecular analysis, while the other half was fixed in 10 % neutral-buffered formalin (NBF) or 4 % paraformaldehyde (PFA) for 24–48 h prior to paraffin embedding and sectioning by standard technique. Paraffin-embedded tissues were serially cross-sectioned on a microtome at a thickness of 7 μm and counterstained with hematoxylin and eosin (H&E) or Masson’s trichrome after deparaffinization and rehydration by standard technique. Slides were imaged and captured on a Nikon TS100 inverted light microscope (Nikon Instruments, Belmont, CA).

### Molecular analysis–quantitative real-time PCR

Mesh explants were separated from native musculature at each time point, homogenized, and lysed using TRIzol reagent (Invitrogen, Carlsbad, CA) followed by DNAse (Sigma-Aldrich, St. Louis, MO) treatment. RNA concentration and purity were measured using a NanoDrop ND1000 spectrophotometer (NanoDrop Technologies, Wilmington, DE). From each sample, cDNA was synthesized from 1 μg total RNA, using the iScript retrotranscription kit (Bio-Rad Laboratories, Hercules, CA). Transcribed products were analyzed using commercially available master mix and the appropriate target probes including: collagen type 3 alpha 1 (*Col3a1*, Rn01437681_m1) and collagen type 1 alpha 1 (*Col1a1*, Rn01463848_m1) to determine collagen deposition, vascular endothelial growth factor A (*Vegf*, Rn01511601_m1), and the von Willebrand factor (*vwf*, Rn01492158_m1) to assess angiogenesis, and alpha 2 smooth muscle actin (*Acta2*, Rn01759928_g1) and fibroblast specific protein 1 (*FSP1*, Rn01451938_m1). Reactions were performed on an ABI 7500 Fast Sequence Detection System (Applied Biosystems, Foster City, CA). At each time point, gene expression of explanted Strattice matrices with PRP (S w PRP) was compared to that obtained from Strattice matrices without PRP (S w/o PRP). Results were normalized to the level of expression of the housekeeping gene glyceraldehyde 3-phosphate dehydrogenase (*GAPDH*; Rn01775763_g1). Three technical replicates for each biologic sample were performed, and results are reported as mean ± standard deviation.

### Statistical analysis

All statistical analysis was performed using GraphPad Instat (GraphPad Software, La Jolla, CA, USA). One-way ANOVA for multiple comparisons by Student–Newman–Keuls test was used for molecular data. For analysis of continuous variables of adhesion severity and residual mesh thickness, comparison was performed using two-tailed, paired *t* tests to directly compare the two groups of interest at each time point and one-way, repeated-measures ANOVA was also used with Tukey’s posttest to compare means across all groups. For all analyses, statistical significance was defined as either: insignificant (*p* > 0.05), significant (*p* ≤ 0.05*), very significant (*p* < 0.01**), or highest significance (*p* < 0.01***).

## Results

**Fig. 2:**
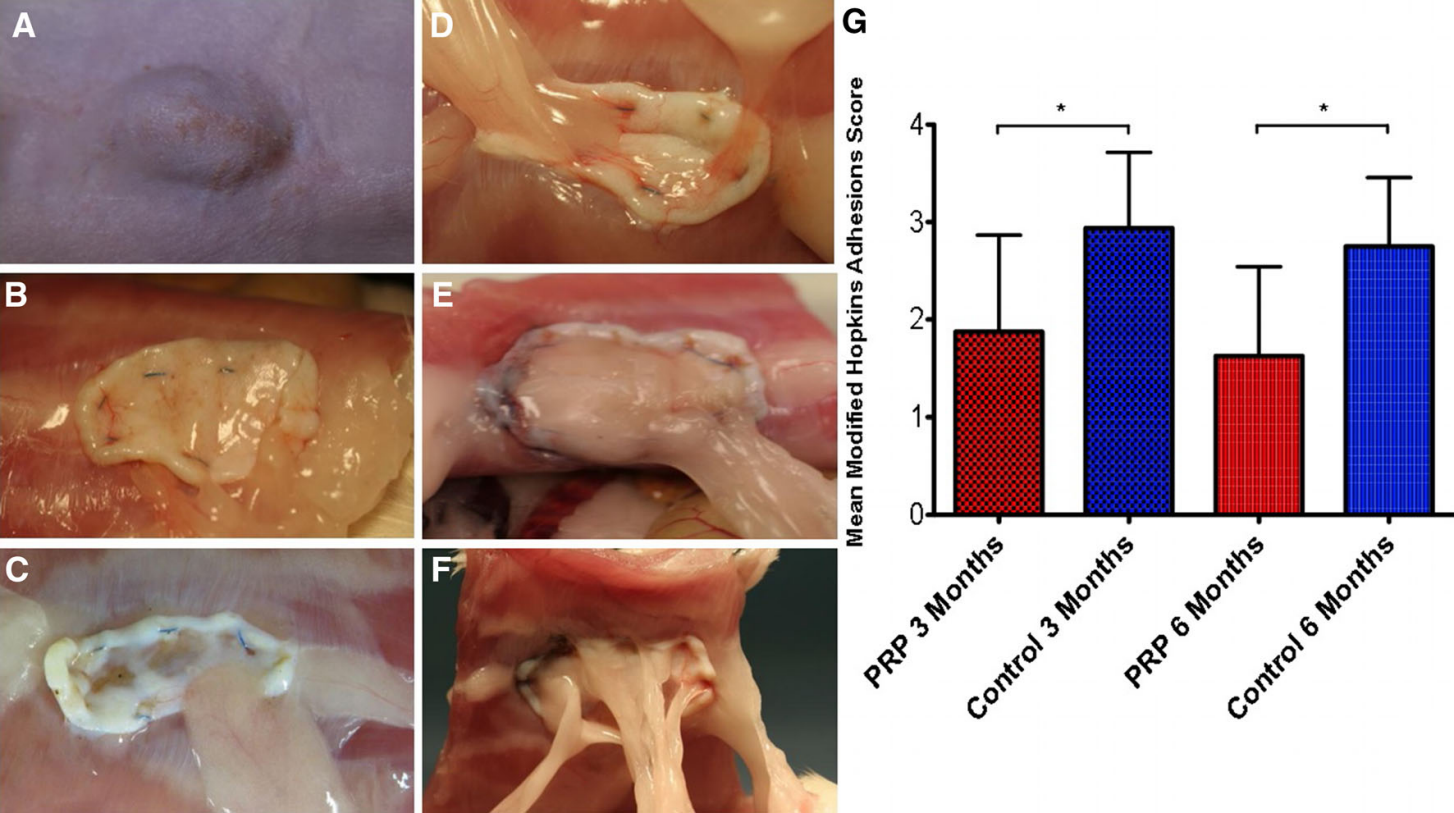
Peritoneal adhesions and Modified Hopkins Adhesion Score. Some control rats displayed obvious external eventration at the time of necropsy evident of underlying hernia recurrence (**A**). Representative images are shown correlating with Adhesion Score of 0 (**B**, PRP+), 1 (**C**, PRP+), 2 (**D**, PRP+), 3 (**E**, PRP−), or 4 (**F**, PRP−). Statistically significant differences were witnessed in mean Modified Hopkins Adhesion Scores between control and experimental rats at both 3 and 6 months (**G**)

Two animals died due to inadvertent general anesthetic complications while operating or in the immediate post-op recovery period and were replaced in the study. No animals suffered wound complications requiring study removal, and the number of clinically significant seromas in each group did not differ significantly—6/18 (33 %) in the control group compared to 5/18 (28 %) in the experimental PRP group. Several distinct differences were noted between experimental groups both at the gross macroscopic level and at the microscopic/molecular level, and also at both time points investigated. Generally speaking, at the time of 3-month necropsy, PRP-treated samples displayed less severe peritoneal adhesions and more readily apparent gross neovascularization of the implanted mesh compared to controls. The mean Modified Hopkins Adhesion Score for the PRP group was 1.88 (±0.99) at 3 months compared to 2.94 (±0.78) in the control group, as assessed according to the parameters outlined in Table 1 and illustrated in Fig. 2—a statistically significant difference (*p* = 0.02). Shown are representative samples for each score 0–4. Only the PRP-treated group had an animal subject that received a score of 0 (no adhesions), while the control group did not have any subjects with a score below 2.0.

**Fig. 3:**
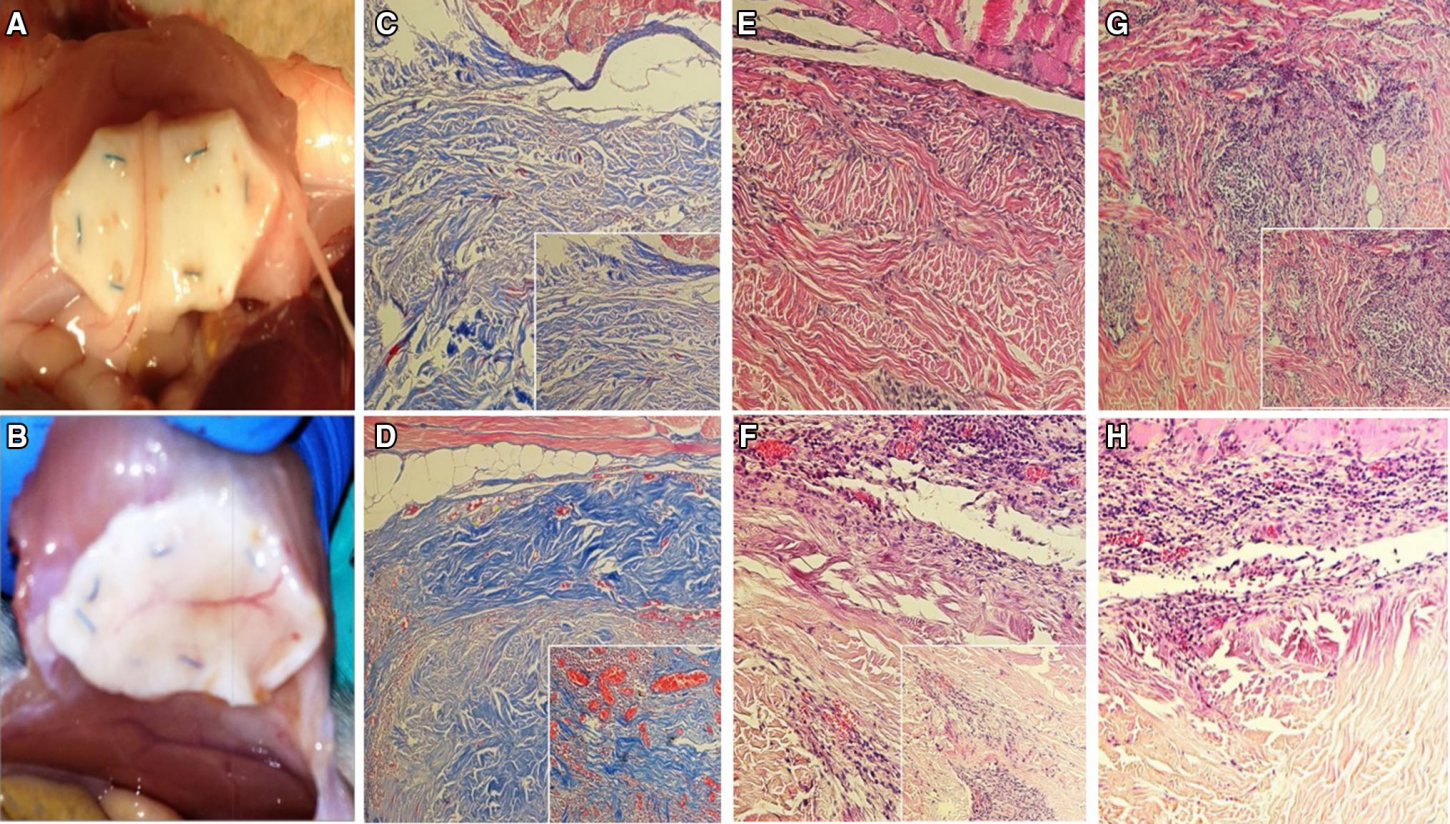
Mesh neovascularization, all images taken at 10× (*large*) or 20× (*inset*) magnification. Significant differences in neovascularization of implanted mesh were noted between experimental groups at the gross level (**A - control**, **B - PRP+**). Histologic analysis of Masson’s trichrome stained specimens confirms this effect, with significant difference in both the size and number of neovessels (*orange*-*red*) and depth of penetration into the mesh (*blue*) of control (**C**) versus PRP-treated (**D**) samples. Additional differences were noted in degree and depth of tissue ingrowth and immune cell reaction as seen in H&E stained specimens. Control samples displayed less ingrowth (**E**) and more chronic inflammatory infiltrate (**G**) compared to PRP-treated samples (**F**, **H**)

**Fig. 4:**
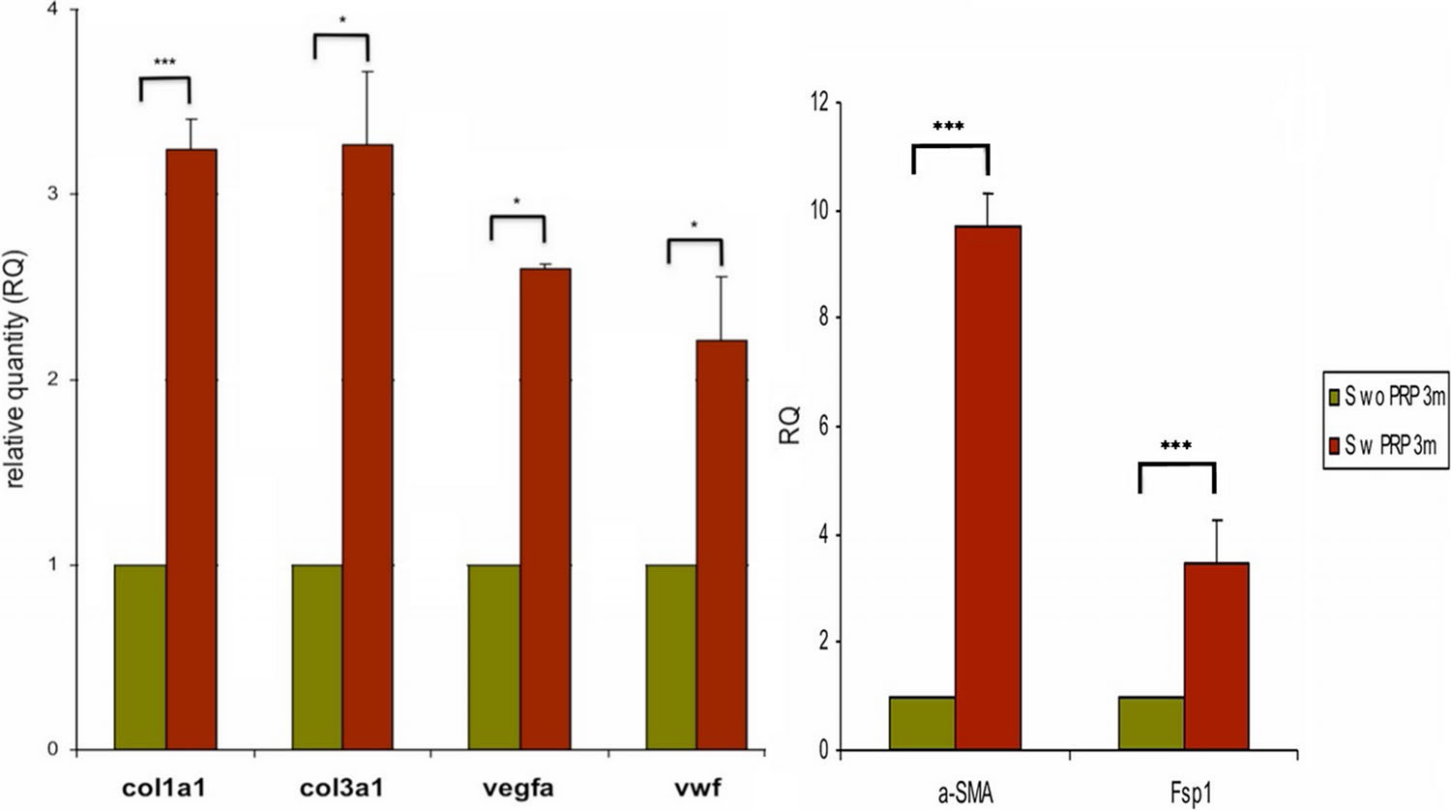
Molecular gene upregulation data. PRP-treated specimens (S w PRP) displayed statistically significant (ranging from *significant to ***highly significant) upregulation of angiogenic genes (vEGFa, vWF), myofibroblastic genes (αSMA, FSP-1), and their biosynthetic products (Col1a1, Col3a1) compared to controls (S wo PRP)

Additionally witnessed at the time of tissue harvest, meshes from the PRP group seemed to have much larger and easily identifiable neovessels infiltrating the implanted mesh compared to controls, as depicted in Fig. 3. This was confirmed histologically, as control animals had small, dispersed neovasculature concentrated mostly at the mesh surface near areas of muscular overlap, correlating with granulation-type tissue. Meanwhile, experimental PRP samples displayed very robust, large, interconnecting networks of neovessels that appeared more mature, originating from areas of tissue overlap but clearly penetrating deeper into the implanted mesh (Fig. 3). This phenomenon was confirmed at the molecular level, as PRP-treated samples demonstrated a significant upregulation of classic genes of angiogenesis compared to controls. At 3 months, a 2.73-fold (±0.09, *p* < 0.05) upregulation was seen for vEGF and a 2.21-fold (±0.38, *p* < 0.05) upregulation for vWF (Fig. 4). Such enhanced angiogenesis appeared linearly connected with improved tissue deposition/ingrowth into the mesh compared to controls (Fig. 3). This was observed in concert with a significant upregulation of genes specific for the presence and activity of fibroblasts/myofibroblasts. The greatest upregulation occurred for αSMA (9.68-fold ± 0.63, *p* < 0.001) and FSP-1 (3.61-fold ± 0.82, *p* < 0.001), but expression of their synthetic collagen products was also heightened—Col31a1 (3.32-fold ± 0.44, *p* < 0.001) and Col1a1 (3.29-fold ± 0.19, *p* < 05, Fig. 4).

**Fig. 5:**
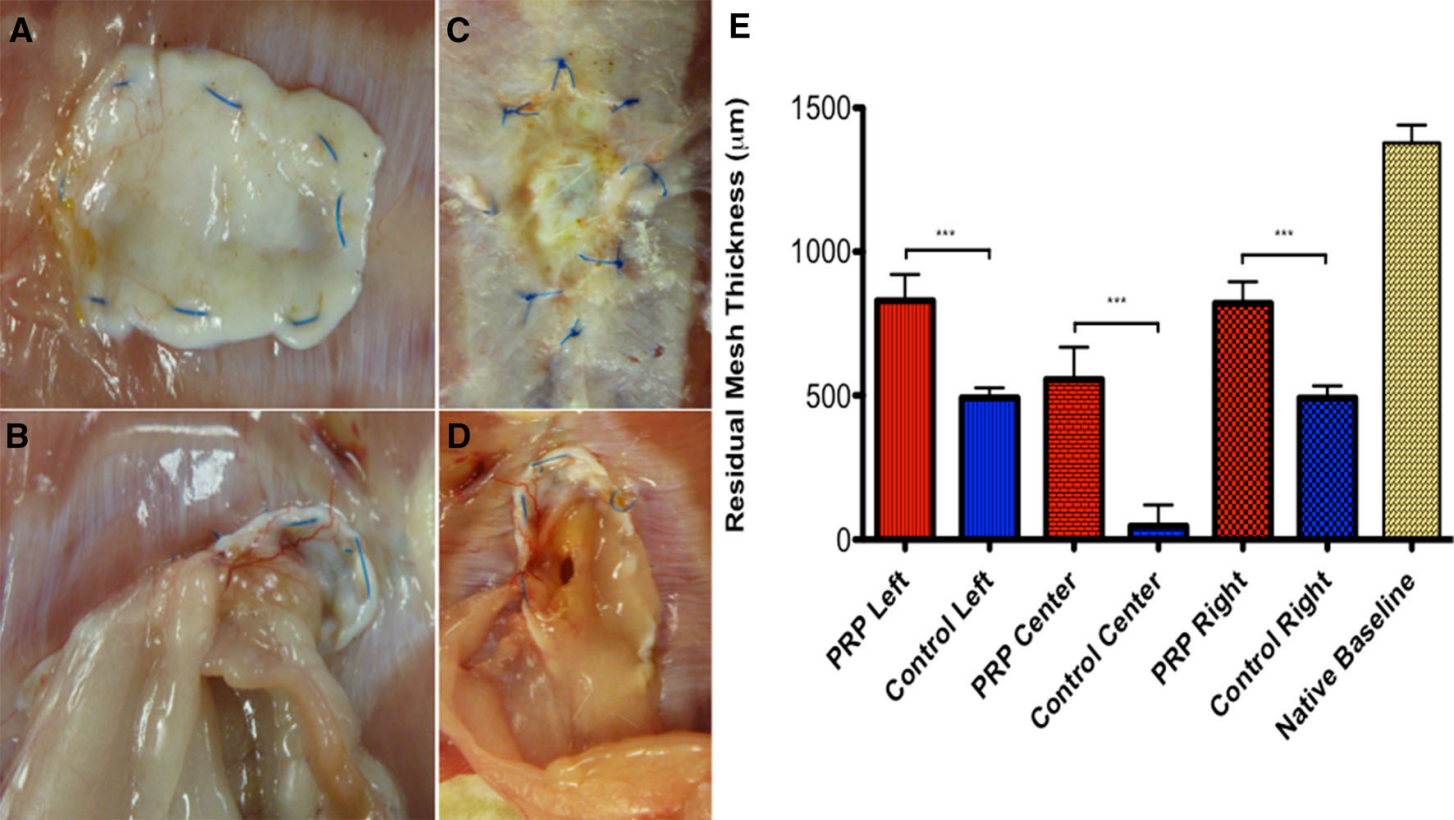
Hernia recurrence and residual mesh thickness. No PRP-treated rats (**A**, **C**) suffered mesh failure and hernia recurrence, while 70 % of control rats recurred (**B**, **D**). Rats invariably recurred at mid-mesh, where the residual prosthesis was thinnest. PRP-treated rats experienced less mesh degradation from baseline and had a thicker preserved ADM remnant at all measured locations than control (**E**)

Impressive differences were also seen at the 6-month endpoint, none more striking than the rate of mesh failure and ventral hernia recurrence. A 70 % recurrence rate (7/10 rats) was encountered in the control group, compared to 0 % in the experimental PRP-treated group. All hernias in control rats recurred at the center of the implanted bridging mesh (Fig. 5). Mesh thickness and architecture were appreciably more preserved in the PRP group at a gross level, and this was confirmed objectively using a digital caliper. Native Strattice™ ADM was measured by caliper at five random locations to obtain a preoperative baseline (1380 μm ± 68.9). At the time of 6-month harvest, specimens had their respective residual ADM similarly measured along the cut edge at the anatomic left, right, and center of mesh. PRP-treated specimens invariably displayed significantly higher mean residual mesh thickness than their control counterparts at all three mesh locations—left (834.9 μm ± 101.0 vs. 482.1 μm ± 28.2, *p* = 0.0006), center (561.3 μm ± 124.1 vs. 56.4 μm ± 78.3, *p* = 0.0004), and right (817.2 μm ± 83.4 vs. 476.1 μm ± 29.7, *p* = 0.0004). The degree of mesh thickness loss from baseline over time in the PRP group was thus 46.5 % compared to 75.5 % in the control group, a remarkable difference (Fig. 5). Finally, differences in peritoneal adhesion severity were seen at 6 months as well, with a decreased mean Modified Hopkins Adhesion Score compared to control (1.63 ± 0.92 vs. 2.75 ± 0.70). As expected from clinical experience, there was a slight trend toward decreasing scores across all groups from 3-month to the 6-month time point.

## Discussion

Our results demonstrate the capability of autologous PRP to enhance cellular metrics of VHR success including neovascularization and tissue ingrowth, resulting over time in diminished hernia recurrence and preserved ADM integrity. The degree of neovascularization is a primary post hoc factor assessed in the literature regarding VHR success with ADM and has been shown to have a direct relationship with tissue incorporation, extent of eventration, and ultimate biomechanical strength [39–43]. We present a multiplicity of objective data confirming enhanced neovascularization of implanted Strattice™ with the addition of PRP, ranging from histologic staining to irrefutable molecular evidence. Though manual neovessel quantification has been performed historically on histologic samples via reproducible field of view (FoV) analysis, the vast majority of prior studies are conspicuously missing similar high-quality molecular comparisons [44]. This is a considerable finding given the amplified propensity of ADMs generally and Strattice™ specifically for neovascularization, even in the baseline control state [37, 39, 45–47]. Like historical studies, we also saw a correlate increase in tissue deposition and ingrowth with the heightened angiogenesis on histologic analysis [48]. We took this a step further to prove that this tissue was of functional cellular phenotype by molecular analysis for genes specific for myofibroblasts and their biosynthetic products of collagen and witnessed a difference of highest statistical significance. This suggests that growth factors/chemokines within a single application of autologous PRP are sufficient to initiate a striking cellular migratory response from the surrounding tissues, and that said cells take up permanent residence within the mesh. Successful cellular recruitment is often elusive after VHR and the notion that such a robust response can be initiated by such a simple measure up front at the time of repair could be transformative for the field of hernia surgery if similar results could be achieved in human application.

Although there is some disagreement in the literature, beyond 3 months postimplantation non-cross-linked ADMs like the one used in this study are believed to undergo more significant degradative thinning compared to cross-linked ADMs despite suggestion of superior incorporation—possibly due to increased sensitivity to enzymatic degradation or chronic inflammatory activation [20, 37, 38, 49]. Adjuvant modalities capable of preserving native ADM architecture and thus biomechanical integrity over time could improve outcomes when ADM is used in bridging repair, and agents that promote concurrent tissue incorporation are even more desirable. Although the exact mechanism(s) by which autologous PRP produces its long-term effect of superior ADM preservation are not entirely clear, it appears preliminarily to represent such an agent capable of parallel advantageous mesh remodeling and protection from degradation. The details of PRP’s effect on immune cell regulation need fleshing out, but the overall clinical effect witnessed in this project of safeguarding from long-term hernia recurrence via mesh failure are striking. We learn from Poulose et al. [5] that by decreasing total VHR operations even a modest 1 % would save the US $32 million, meaning that any method of decreasing hernia recurrence can have a tremendous economic impact atop its inherent clinical benefit. The decrease witnessed in severity of peritoneal adhesions was curious and must be further proven in subsequent studies, but may again be related to effects on the native immune response. If PRP can in fact diminish adhesion incidence and severity, this would have significant translational implications since adhesive disease is the most common cause of small bowel obstruction requiring hospitalization, and sometimes even surgical treatment [50, 51]. Taken together, these results warrant, at a minimum, investigation in subsequent animate models and preliminary study in human VHR. The potential clinical implications of autologous PRP application range from standardized implementation of PRP with all VHR to more routine use of biologic ADMs for definitive VHR, and even to expanded uses of ADM in breast/burn surgery or other clinical scenarios of soft tissue wound healing.

Every study has its limitations, and though we are excited by our results above, we realize that they must be viewed through an objective lens of how future studies might be improved. Although the significance of the difference between our groups was previously unknown, it proved sufficient enough to produce statistically relevant data; yet we would have liked to expand the numbers of subjects and time points analyzed but were limited by the availability and financial burden of the Strattice™ ADM used. Larger group sizes would avoid the potential of making a type 1 error and concluding a false-positive difference by rejecting the null hypothesis. The addition of early postoperative time points may help clarify the true mechanism of PRP’s effect at the cellular level and on the native immune system. This is an area of particular interest to our laboratory that may hold the keys to unlocking the ultimate potential of autologous growth factors and we plan to investigate further. Additionally, the immunobiologic milieu of the rodent is undoubtedly different than that of a human and, since that very milieu is a central component to either the successful incorporation or degradation/rejection of a hernia mesh generally speaking and within a biologic ADM specifically, more advanced animate testing that more closely mimics humans is necessary before drawing immediate conclusions. Such porcine and primate models exist in the literature and have never been investigated regarding PRP’s utility, but are fairly cost-prohibitive [8, 52–54]. An experienced veterinary histopathologist was not available for review and scoring of our histologic samples, and although histologic outcome differences were not primary endpoints of this study, having such experienced input would bring objective value to our anecdotally noted differences in regards to tissue incorporation and immunologic cell infiltration. Cell- or tissue-specific histologic staining may also be helpful to support our striking molecular data to visually demonstrate the presence of myofibroblasts and/or collagen to a greater degree, as well as proving the presence and specific phenotype of immune cells present. These will be incorporated in future studies.

Finally, we were surprised at the higher than anticipated incidence of hernia recurrence witnessed in our 6-month control group, but a host of factors likely contributed to variable degrees. The size of the hernia defect in the rat was very large in relation to body habitus and correlates with what would be a massive hernia defect in the human. The implanted mesh experiences increased stress at baseline as a bridging repair; and the quadruped nature of the rat along with their increased body weight after hernia creation and repair add additional tension to the mesh center, a primary factor contributing to VHR failure [8, 55, 56]. It is a known fact that ADMs have a higher recurrence rate when used in a bridging repair compared to reinforcing a primary repair [55]. However, we controlled well for PRP application by keeping all other factors the same (hernia defect and mesh implant size, surgical technique, average rat size), and thus, one would expect similar rates of mesh degradation and failure in the experimental group if PRP did not have a significant impact. Adding mechanical strain testing of our explanted samples would add further objective, comparative data, and we plan to incorporate this with repeat studies which are already underway.
